# Hematotoxic Effect of Respiratory Exposure to PHMG-p and Its Integrated Genetic Analysis

**DOI:** 10.3390/toxics10110694

**Published:** 2022-11-16

**Authors:** Hwa Jung Sung, Sang Hoon Jeong, Ja Young Kang, Cherry Kim, Yoon Jeong Nam, Jae Young Kim, Jin Young Choi, Hye Jin Lee, Yu Seon Lee, Eun Yeob Kim, Yong Wook Baek, Hong Lee, Ju Han Lee

**Affiliations:** 1 Department of Oncology and Hematology, Ansan Hospital, Korea University College of Medicine, Ansan-si 15355, Gyeonggi, Republic of Korea; 2 Medical Science Research Center, Ansan Hospital, Korea University College of Medicine, Ansan-si 15355, Gyeonggi, Republic of Korea; 3 Department of Radiology, Ansan Hospital, Korea University College of Medicine, Ansan-si 15355, Gyeonggi, Republic of Korea; 4 Humidifier Disinfectant Health Center, Environmental Health Research Department, National Institute of Environmental Research, Incheon 22689, Republic of Korea; 5 Department of Pathology, Ansan Hospital, Korea University College of Medicine, Ansan-si 15355, Gyeonggi, Republic of Korea

**Keywords:** polyhexamethylene guanidine phosphate (PHMG-p), respiratory exposure, hematopoietic toxic effect, bone marrow (BM), Ingenuity Pathway Analysis (IPA)

## Abstract

Polyhexamethylene guanidine phosphate (PHMG-p), the main ingredient of humidifier disinfectants, circulates systemically through the lungs; however, its toxicological assessment has been primarily limited to pulmonary disease. Herein, we investigated the possible abnormalities in hematopoietic function 20 weeks after intratracheal instillation of PHMG-p in a rat model. Notable abnormalities were found out in the peripheral blood cell count and bone marrow (BM) biopsy, while RNA sequencing of BM tissue revealed markedly altered gene expression. Furthermore, signaling involved in hematopoietic dysfunction was predicted by analyzing candidate genes through Ingenuity Pathway Analysis (IPA) program. Respiratory PHMG-p exposure significantly decreased monocyte and platelet (PLT) counts and total protein, while significantly increasing hemoglobin and hematocrit levels in peripheral blood. Histopathological analysis of the BM revealed a reduced number of megakaryocytes, with no significant differences in spleen and liver weight to body weight. Moreover, PHMG-p exposure significantly activated estrogen receptor signaling and RHOA signaling, and inhibited RHOGDI signaling. In IPA analysis, candidate genes were found to be strongly related to ‘hematological system development and function’ and ‘hematological disease.’ Accordingly, our results suggest that PHMG-p could affect hematopoiesis, which participates in monocyte differentiation and PLT production, and may induce hematologic diseases via the respiratory tract.

## 1. Background

Polyhexamethylene guanidine phosphate (PHMG-p) is known to exert antiseptic and sterilizing effects and was widely used in household humidifier disinfectants (HDs) in South Korea. Following an outbreak of patients with unexplained pneumonia, respiratory failure, and mortality in 2002, nationwide epidemiological studies have reported the presence of PHMG-p in HD-induced lung fibrosis and respiratory failure [1,2,3]. Subsequent investigations have attempted to elucidate the mechanisms through which PHMG-p causes pulmonary alveolar damage and lung interstitial fibrosis from toxicological and immunological perspectives using a PHMG-p-exposed rat model [4]. However, the systemic toxic effects of PHMG-p in other organs remain unexplored. Once PHMG-p is released into the air and inhaled, it becomes a fine particle, penetrating the alveolar-capillary barrier and entering the systemic circulation. Theoretically, this chemical could induce systemic effects which remain unknown [5]. Few animal studies have reported the systemic toxic effects of inhaled PHMG-p. Kim et al. have shown that HDs can induce acute cardiovascular toxic effects, heart failure caused by severe inflammation, atherogenesis, and aging, accompanied by embryonic toxicity in a zebrafish model [6]. However, the effect of inhaled PHMG-p on hematopoiesis needs to be explored.

Abnormalities in the hematopoietic process via respiratory exposure to chemicals have been previously reported. Benzene is a highly flammable volatile hydrocarbon solvent that is widely employed as an industrial chemical. Respiratory exposure to benzene can cause leukemia; hence, benzene is classified as a carcinogen, given to its bone marrow (BM)-induced toxicity [7,8]. In addition, airborne particulate matter (PM) can reach the bloodstream through alveoli and cause widespread toxicity through systemic circulation [9]. Exposure to PM causes hematopoiesis-related abnormalities, such as leukocytosis and alterations in the number of neutrophils and monocytes [10,11]. Dioxins are unnecessary by-products released into the atmosphere following the incineration of solid waste [12]. Upon activation, dioxin can induce hematopoietic toxicity by interacting with the aryl hydrocarbon receptors expressed in BM tissue and lymphoid stem cells [13]. These compounds and their metabolites could directly affect the BM by interfering with hematopoietic pathways associated with the differentiation and maturation of progenitor cells. This process, in turn, leads to failure of the BM microenvironment, resulting in a reduction in peripheral blood cell counts, such as erythrocytes, leukocytes, and platelets (PLTs), thereby inducing pancytopenia. Moreover, hematological disorders, such as aplastic anemia, myelodysplastic syndrome, and acute leukemia, may develop gradually.

In the present study, we investigated the effect of PHMG-p on the hematopoietic system following intratracheal PHMG-p instillation in a rat model and identified alterations in gene transcription in BM tissues.

## 2. Methods and Materials

### 2.1. Animals

This study was approved by the Institutional Animal Care and Use Committee of Korea University Medical Center (approval number: Korea-2021-0051). All experiments were performed according to the National Institutes of Health Guide for the Care and Use of Laboratory Animals, and all experiments were conducted in accordance with the Korea University guidelines. Nine-week-old male Sprague–Dawley rats (Raonbio, Yong-in, South Korea) were acclimatized for one week under the following conditions: temperature, 22–25 °C; relative humidity, 40–60%; and lighting conditions, light 12 h/dark 12 h. Pelleted food (Purina, Sung-nam, South Korea) for experimental rodents and filtered tap water were provided ad libitum. During the experimental period, the weight change, appearance, measurable clinical features, and behavioral responses to external stimuli were assessed weekly by specialized facility staff. The femur and tibia of rats were harvested under anesthesia following intraperitoneal and intramuscular injections of alfaxan (30 mg/kg) and xylazine (10 mg/kg), respectively. Rat BM was collected according to an internal protocol.

### 2.2. Experimental Design

Thirty rats were randomly divided into two groups. A solution of PHMG-p was diluted to 0.9 mg/kg using saline, as described previously [14]. Rats were anesthetized with 2% isoflurane in 70% N_2_O and 30% O_2_ for intratracheal instillation of PHMG-p. Normal saline and PHMG-p solution (50 µL) were intratracheally instilled into corresponding group of rats five times at two-week intervals. PHMG-p solution was administered under the guidance of a modified videoscope. After intratracheal instillation of PHMG-p at 0, 2, 4, 6, and 8 weeks, all rats were sacrificed at 20 weeks after the first instillation.

### 2.3. Blood Samples

After sacrificing experimental rats, approximately 3 mL of blood was collected from the aorta of each rat. Blood samples were collected into K2 EDTA 5.4 mg blood collection tubes (BD, Franklin Lakes, NJ, USA), and white blood cells (WBCs; band neutrophils, lymphocytes, monocytes, eosinophils, and basophils), red blood cells (RBCs), and PLTs were analyzed using ADVIA 2120 (Siemens, Munich, Germany). Total protein, albumin, creatinine, C-reactive protein (CRP), and blood urea nitrogen (BUN) levels were analyzed using an automated machine Cobas 6000 (Roche, Basel, Switzerland).

### 2.4. Reagents

PHMG-p was obtained from BOC Sciences (Shirley, NY, USA; CAS registry number 89697-78-9). Normal saline was obtained from the JW Pharmaceutical Co. (Seoul, Korea). TRIzol^TM^ reagent (#15596026) and diethyl pyrocarbonate (DEPC)-treated water (AM9906) were purchased from Thermo Fisher Scientific (Waltham, MA, USA). Chloroform (C2432-25ML), ethyl alcohol (E7023-1L), and 2-propanol (278475-250ML) were obtained from Sigma-Aldrich (St. Louis, MO, USA).

### 2.5. Histologic and Immunohistochemical Examination

The harvested rat femurs were fixed in 10% neutral buffered formalin for 24 h. For decalcification, femurs were placed in 10% nitric acid for 8 h. Then, 4-µm thick paraffin sections were cut from the fixed samples, following staining with hematoxylin and eosin (H&E). For immunohistochemical staining, representative tissue sections were deparaffinized and dehydrated, and heat pre-treatment was performed for 20 min. After incubation with peroxide for 10 min, the slides were stained with an antibody against integrin β3 (clone D-11, 1:200; Santa Cruz, TX, USA). After incubation with secondary antibodies at room temperature for 10 min, the sections were developed with 3, 3′-diaminobenzidine and counterstained with Harris hematoxylin. To measure the number of megakaryocytes, H&E staining and integrin immunostaining were used to count the number of megakaryocytes in 10 consecutive locations at 400× magnification.

### 2.6. RNA Isolation

Total RNA was isolated using TRIzol reagent, according to the manufacturer’s instructions. RNA quality was assessed using an Agilent 2100 Bioanalyzer with the RNA 6000 Nano Chip (Agilent Technologies, Amstelveen, The Netherlands), and RNA samples were quantified using an ND-2000 spectrophotometer (Thermo Inc., Waltham, MA, USA).

### 2.7. Library Preparation and Sequencing

The library was constructed from total RNA using the NEBNext^®^ Ultra^TM^ II Directional RNA-Seq Kit (NEW ENGLAND BioLabs Inc., Ipswich, MA, USA). Ribosomal RNA (rRNA) was eliminated using the RiboCop rRNA depletion kit (LEXOGEN Inc., Vienna, Austria). RNAs that did not contain rRNA were used for cDNA synthesis and shearing in accordance with the manufacturer’s instructions. Indexing was performed using Illumina indexes 1–12. Enrichment was performed using polymerase chain reaction (PCR). Subsequently, libraries were verified using the Agilent 2100 Bioanalyzer (DNA High Sensitivity Kit) to assess the average fragment size. Quantification was performed using a library quantification kit on a StepOne Real-Time PCR System (Life Technologies Inc., Carlsbad, CA, USA). QuantSeq 3′-mRNA sequencing was performed as paired-end 100 sequencing using NovaSeq 6000 (Illumina Inc., San Diego, CA, USA).

### 2.8. Data Analysis

Quality control of the raw sequencing data was performed using FastQC [15]. Low-quality reads (<Q20) and adapters were eliminated using FASTX_Trimmer [16] and BBMap [17]. The trimmed reads were then mapped to the reference genome using TopHat [18]. Gene expression was estimated using fragments per kilobase per million reads (FPKM) using Cufflinks [19]. The FPKM values were normalized based on the quantile normalization method using EdgeR in R [20]. On analyzing the top canonical pathways for candidate genes, the length of bars was expressed based on Fisher’s exact test *p*-value. The criterion for the score cutoff is an item with a −log(*p*-value) > 1.3 and an absolute *z*-score > 0. The color of the canonical pathway graph bar indicates the *z*-score. Hematopoiesis-related diseases and signaling pathways were deduced using Ingenuity Pathway Analysis (IPA) program (Qiagen, MD, USA).

### 2.9. Statistical Analyses

All data were analyzed using GraphPad Prism v.5.0 (GraphPad Software, CA, USA) and are expressed as mean ± standard deviation (SD). IBM SPSS/WIN v25.0 (IBM Corp., Armonk, NY, USA) was used for statistical analysis, and the statistical significance level was set at *p* < 0.05. In a statistical analysis of 15 rats per group for a total of 30 rats, the PLT count, megakaryocyte count, liver weight, body weight, and liver/body weight were analyzed using the *t*-test, while the spleen weight and spleen/body weight were analyzed using the Mann–Whitney U test.

## 3. Results

### 3.1. Altered Blood Composition Induced by PHMG-p Respiratory Exposure

To investigate the hematotoxic effect of PHMG-p respiratory exposure, PHMG-p was intratracheally instilled. The exposure concentration of PHMG-p was 1 mg/kg for each instillation, for a total of 5 mg/kg, thereby simulating the use of HDs for 6 h per day for 5 months in a room of 30 m^3^ volume and ventilation rate of 0.2.

The mean values, SD, and *p*-values for blood test results of PHMG-p-exposed and normal saline groups (*n* = 15 rats/group) for a hematotoxic total of 30 animals are listed in Table 1, Table 2 and Table S1. The average PLT count was 928.7 ± 142.7 × 10^3^/µL and 703.1 ± 180.0 × 10^3^/µL in the normal saline and experimental groups, respectively. The megakaryocyte count decreased in the PHMG-p-exposed group, but the decrease was not statistically significant (Table S1). The average spleen weight was significantly reduced by 1.061 ± 0.287 g and 0.779 ± 0.114 g in the normal saline and the PHMG-p-exposed groups, respectively; however, no significant differences were noted in liver and body weights (Table S1). In case of WBCs, the proportion of monocytes was 3.50 ± 0.73% and 2.40 ± 0.88% in the normal saline and PHMG-p-exposed groups, respectively, with statistically significant difference noted between the two groups (*p* = 0.003; Table 1). Hematocrit (Hct), the concentration of RBCs in total blood, was significantly increased in the PHMG-p-exposed group (*p* = 0.024). These findings could be attributed to a compensatory mechanism to counteract the decreased oxygen exchange capacity induced by lung injury. The percentage mean of band neutrophils (normal saline: 32.60 ± 18.56%, PHMG-p: 41.24 ± 21.15%) and basophils (normal saline: 1.23 ± 0.89%, PHMG-p: 2.03 ± 1.56%) was increased in the PHMG-p-exposed group, although no statistical significance was noted. Despite differences in total serum protein between both groups (6.25 ± 0.51 g/dL and 5.79 ± 0.31 g/dL, respectively), both values were within the mean ± SD values of each other with significance (*p* = 0.012). Total protein concentration in peripheral blood was significantly decreased in the group exposed to PHMG-p (*p* = 0.012) (Table 2). However, albumin (normal saline: 3.87 ± 0.38 g/dL, PHMG-p: 3.74 ± 0.16 g/dL) belonging to total protein was not statistically significant (*p* = 0.439). BUN (normal saline: 22.07 ± 3.73 mg/dL, PHMG-p: 26.40 ± 7.60 mg/dL) and creatinine (normal saline: 0.59 ± 0.14 mg/dL, PHMG-p: 0.67 ± 0.13 mg/dL) also indicated no statistical significance with the values (*p* = 0.113 and *p* = 0.135, respectively) (Table 2). We examined the homogeneity of variance and applied the *t*-test or Mann–Whitney U test to each item depending on the observed results.

The average PLT count was 928.7 ± 142.7 × 10^3^/µL in the normal saline group and 703.1 ± 180.0 × 10^3^/µL in the PHMG-p-exposed group, indicating a statistically significant difference in PLT count between the two groups (*p* = 0.001). The mean number of PLT-releasing megakaryocytes in the BM was 173.0 ± 31.99 and 151.1 ± 30.40 per 10 high-power fields (HPF) in the normal saline and PHMG-p-exposed groups, respectively (*p* = 0.064; Table S1). PHMG-p exposure significantly impacted the spleen weight to body weight ratio, with values of 0.0021 ± 0.0006 and 0.0015 ± 0.0002 in the normal saline and PHMG-p-exposed groups, respectively (*p* = 0.007). The liver/body weight ratios were 0.0360 ± 0.0061 and 0.0313 ± 0.0048 in the normal saline and PHMG-p-exposed groups, respectively (*p* = 0.026) (Table S1). Given that the organ-to-body weight ratio was reduced in the PHMG-p-instilled group, it is unlikely that increased PLT clearance may have influenced the decrease in PLT count.

### 3.2. Number of Megakaryocytes in PHMG-p-Exposed Group Selected Based on Decreased PLT Count

**Figure 1:**
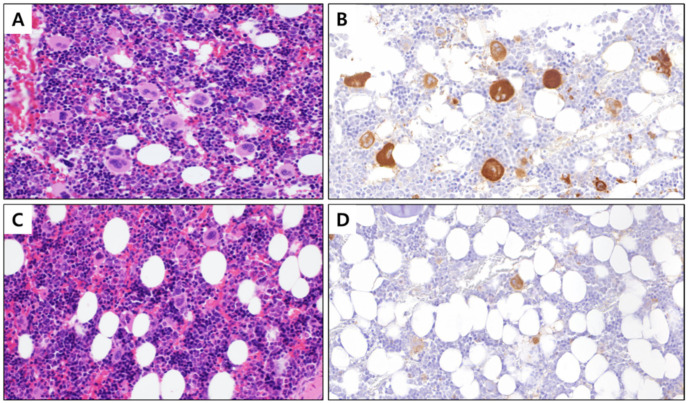
BM tissue with stained megakaryocytes (400×). (**A**) Control BM stained with hematoxylin and eosin, (**B**) control BM with megakaryocyte cytoplasm immunostained with integrin β3, (**C**) PHMG-p exposed BM stained with hematoxylin and eosin, and (**D**) PHMG-p-exposed BM with the cytoplasm of megakaryocytes immunostained with integrin β3. BM, bone marrow; PHMG-p, polyhexamethylene guanidine phosphate.

Given the significant reduction in PLTs in the PHMG-p-exposed group (*p* = 0.001), we selected five animals per group, which exhibited the highest differences between groups based on the PLT count (Table 3). On re-analyzing the blood test items of selected subjects, the PLT count decreased by approximately 2-fold in the PHMG-p-exposed group, with values of 1068.6 ± 69.16 and 503.4 ± 112.4 × 10^3^/µL, respectively, in the normal saline and PHMG-p-exposed groups (*p* = 0.009). Furthermore, we confirmed that the number of megakaryocytes decreased significantly, presenting values of 204.4 ± 33.49 and 133.8 ± 40.33 counts per 10 HPF in the normal saline and PHMG-p-exposed groups, respectively (Figure 1). However, we noted no significant differences (*p* = 0.251) in the spleen and liver weights when compared with the body weight (Table 3). Based on these findings, we predicted that PHMG-p would affect the development of PLT-producing megakaryocytes; therefore, we next examined the related genes in the BM. The BM was collected from the femur and tibia of five selected animals per group.

### 3.3. Identification of Altered Genes Expression in BM with Reduced Megakaryocytes

Collected BM samples were subjected to RNA sequencing. Among analyzed genes, candidate genes were selected based on the criteria of more than double-fold expression between groups, *p*-value < 0.05, and normalized data (log2) value ≥ 4.0. Based on these criteria, 67 upregulated and 75 downregulated genes were identified, with no significant differences in the number of genes between increased and decreased gene candidates (Table 4). However, we found a significant difference in the absolute value of gene expression fold change (FC) between upregulated and downregulated genes. If the criterion for a significant FC difference was set to 5.0, the number of genes with increased expression was counted as 9, and the number of genes with decreased expression was counted as 27 (Table 4).

In particular, PHMG-p exposure induced the expression of Txk, Ctnnal1, Ctsw, Hfe, Arhgap4, Lactb, Clcc1, Tyk2, and Mrps23, which were closely related to hematopoietic signaling in the IPA dataset. Furthermore, the expression of Rho GTPase-activating protein 4 (Arhgap4), a GTPase-activating protein (GAP) coding gene, was increased by 7.775-fold in the BM of the PHMG-p-exposed group. Furthermore, the expression of Rho GTPase activating protein 11A (Arhgap11a) was upregulated by 2.892-fold. Consistent with this finding, the expression of Rho guanine nucleotide exchange factor 3 (arhgef3), encoding the guanine nucleotide exchange factor (GEF) protein, which plays a contradictory role to GAP in the morphological change of megakaryocytes for PLT production, decreased by 7.7-fold in the PHMG-p-exposed group. Surprisingly, hematopoietic prostaglandin D synthase (Hpgds), the most downregulated in the PHMG-p-instilled group (FC: −20.212, *p*-value: 0.036), is a known megakaryocytic-erythroid progenitor (MEP)-specific marker [24].

### 3.4. Predicted Top Canonical Pathways Significantly Related to Candidate Genes

**Figure 2:**
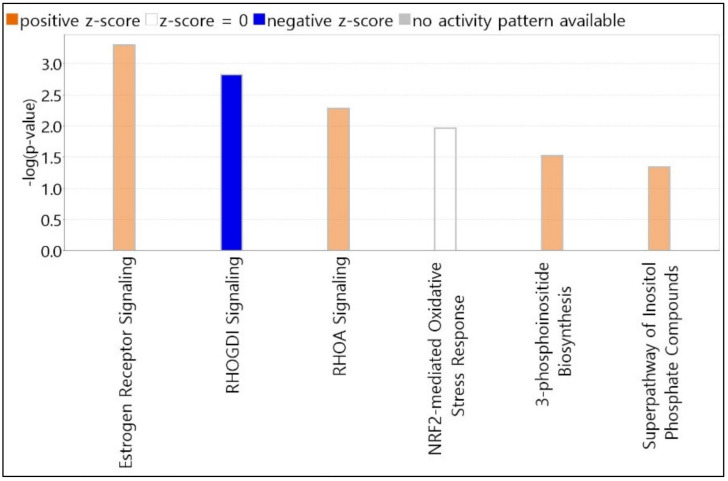
Top canonical pathways elicited by PHMG-p exposure. Pathway candidates selected using IPA core analysis. All canonical pathways registered on the IPA dataset were automatically matched and analyzed for candidate genes. The length of bars is indicated based on the *p*-value of Fisher’s exact test. IPA, Ingenuity Pathway Analysis; PHMG-p, polyhexamethylene guanidine phosphate.

We selected the top 6 canonical pathways using the IPA core analysis. All canonical pathways registered on the IPA dataset were automatically matched and analyzed for candidate genes. The length of bars was indicated based on the *p*-value of Fisher’s exact test. The score cutoff is displayed only for entities with a −log(*p*-value) > 1.3 and an absolute value of *z*-score > 0. The color of the graph bar was expressed based on the *z*-score. Positive *z*-scores are shown in orange, and negative *z*-scores are indicated in blue. The *z*-score uses patterns of protein phosphorylation and gene expression in downstream cascades to predict the activation state of respective upstream regulators. Analyzing our candidate genes using the IPA program, all items with *z*-score calculated as NaN (Not a Number) were excluded. As shown in Figure 2, nine molecules (MED13, NCOR1, GPS2, LIMK2, TYK2, MMP9, CREBBP, PLCL2, and ATP5F1A) were associated with estrogen receptor signaling, with a *z*-score = 1.000 and −log(*p*-value) = 3.296. RHOGDI and RHOA signaling pathways contained six molecules (PIP5K1C, LIMK2, CREBBP, ARHGEF3, ARHGAP4, and PIP5K1B) with a *z*-score = −1.633 and −log(*p*-value) = 2.818, and four molecules (PIP5K1C, LIMK2, ARHGAP4, and PIP5K1B) with a *z*-score = 1.000 and −log(*p*-value) = 2.28. Five molecules (CREBBP, PPIB, GSTZ1, FMO1, and JUNB) were associated with NRF2-mediated oxidative stress response signaling, with a *z*-score = 0.000 and −log(*p*-value) = 1.963. 3-Phosphoinositide biosynthesis and superpathway of inositol phosphate compounds contained 4 molecules (PIP5K1C, UBLCP1, PTPN12, and PIP5K1B) with *z*-scores = 1.000 and 1.000, and −log(*p*-values) = 1.521 and 1.347, respectively.

### 3.5. Predicted Top Diseases and Biological Functions Associated with Candidate Genes

The top five subcategories involving gene candidates in three large categories, i.e., ‘disease and disorder’, ‘physiological system development and function’, and ‘molecular and cellular functions’, were identified (Table 5). In the first parent category, the top two putative ‘disease and disorder’ significantly associated with candidate genes consist of ‘cancer’ and ‘organismal injury and abnormalities’, comprising ‘non-hematological solid tumor (*p* = 4.98 × 10^−8^, 126 molecules)’ and ‘tumorigenesis of tissue (*p* = 5.95 × 10^−8^, 125 molecules)’ in common. The third ‘gastrointestinal disease’ composed of ‘intestinal carcinoma (*p* = 6.60 × 10^−6^, 100 molecules)’ and ‘hepatocellular carcinoma (*p* = 2.70 × 10^−4^, 26 molecules)’. In particular, ‘hematological disease’ consisted of ‘abnormal morphology of bone marrow’ and ‘bone marrow neoplasm’ with a high *p*-value (*p* = 1.30 × 10^−4^ and 7.41 × 10^−4^, respectively), despite the small number of associated molecules.

In the upper category, i.e., ‘physiological system development and function’, the hematopoietic item ‘hematological system development and function’ was also included. The item consisted of ‘morphology of lymphoid tissue (*p* = 4.49 × 10^−5^, 15 molecules)’ and ‘leukopoiesis (*p* = 1.28 × 10^−4^, 19 molecules)’. The third parent category, ‘molecular and cellular functions’, comprised ‘cellular development’ which consisted ‘cell proliferation of tumor cell lines (*p* = 1.61 × 10^−4^, 34 molecules)’ and ‘development of hematopoietic cells (*p* = 4.23 × 10^−4^, 9 molecules)’ (Table 5).

### 3.6. Top Five Annotations Related to Hematological Diseases and Biofunctions

Candidate genes were mapped using IPA, and the top five specific diseases and biological functions corresponding to ‘hematological disease’ and ‘hematological system development and function’ are listed in Table 6. Subgroup annotations belonging to ‘hematological disease’ were sorted according to *p*-value, and the top five significant items were accompanied by ‘abnormal morphology of bone marrow (*p* = 1.30 × 10^−4^, 5 molecules: CREBBP, EBF1, EP400, KAT6A, and TYK2)’, ‘bone marrow neoplasm (*p* = 7.41 × 10^−4^, 28 molecules: ATP5F1A, ATP6V1E1, CCNL1, CD74, CKS1B, CREBBP, CTSW, DHX15, DTNB, EDC4, EP400, IPO7, JUNB, KAT6A, KMT2E, LY6E, LYPD3, MMP9, NCOR1, PDE4B, PLAGL2, PLCL2, POLB, PSMD9, TAF1, TYK2, UBE2I, and ZFP64)’, ‘central nervous system leukemia (*p* = 1.09 × 10^−3^, 2 molecules: MMP9, POLB)’, ‘T acute lymphoblastic leukemia (*p* = 1.35 × 10^−3^, 6 molecules: CREBBP, EBF1, NCOR1, POLB, TAX1BP1, and TYK2)’, and ‘incidence of lymphoma (*p* = 1.74 × 10^−3^, 6 molecules: CD74, DMTF1, INO80, JUNB, MXI1, and TYK2)’ (Table 6).

The top five details corresponding to ‘hematological system development and function’ are listed as ‘morphology of lymphoid tissue (*p* = 4.49 × 10^−5^, 15 molecules: BRD4, CD74, CKS1B, CREBBP, GSTZ1, HPGDS, IRF7, KAT6A, KMT2E, MXI1, NCOR1, SLC35C1, SNX27, ST3GAL2, and TYK2)’, ‘leukopoiesis (*p* = 1.28 × 10^−4^, 19 molecules: C1QC, CD74, CREBBP, DMTF1, EBF1, EIF6, GPS2, IRF7, JUNB, KMT2E, NCOR1, NRROS, PIP5K1C, PLCL2, POLB, PTPN12, RHEB, TXK, and TYK2)’, ‘development of hematopoietic cells (*p* = 4.23 × 10^−4^, 9 molecules: CD74, CREBBP, EBF1, EIF6, KMT2E, MMP9, NCOR1, RCOR1, and TYK2)’, ‘conjugation of T lymphocytes (*p* = 6.41 × 10^−4^, 2 molecules: PIP5K1C and TXK)’, and ‘quantity of erythroid cells (*p* = 1.36 × 10^−3^, 2 molecules: CREBBP and GPS2)’ (Table 6).

### 3.7. Schematic of Hematopoietic Signaling Pathways and Predicted Key Molecules via IPA

**Figure 3:**
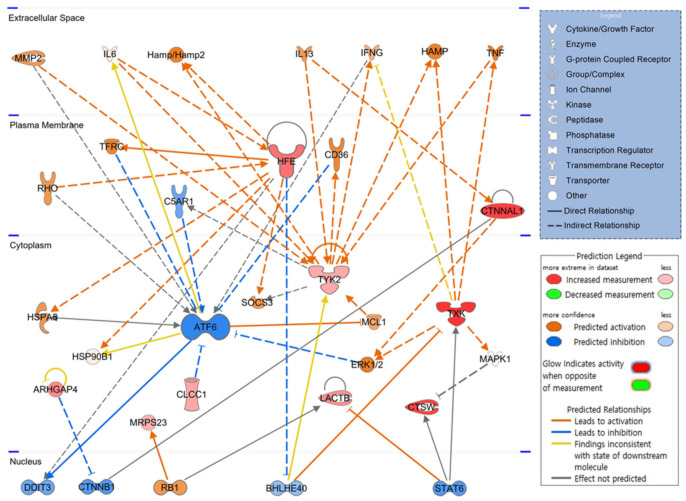
Core signaling pathways related to hematopoiesis. Twenty weeks after intratracheal instillation of PHMG-p, molecules associated with candidate genes analyzed in the bone marrow are schematized as a hematopoietic signaling pathway. PHMG-p, polyhexamethylene guanidine phosphate.

To elucidate how candidate molecules interact and influence hematopoiesis-related signaling, up or downregulated gene candidates were automatically compared and analyzed on the extensive molecular network available in the Ingenuity Knowledge Base (over 100,000 curated libraries). The signaling pathway related to hematopoiesis was visualized based on the interrelationships between candidate molecules. In the expected top-scoring signaling pathway shown in Figure 3, the red figure indicates the increase in molecules, the green represents the decrease, the orange indicates the activation of the corresponding molecule, and the blue figure illustrates inhibited activity. As shown in the schematic network diagram using a part of candidate genes, expression of HFE and CTNNAL, upregulated by PHMG-p, increased in the plasma membrane, and expression of TXK, CTSW, ARHGAP4, LACTB, CLCC1, TYK2, and MRPS23 increased in the cytoplasm. ATF6, not included in the candidate gene list, is expected to be inactivated by CLCC1, TFRC, C5AR1, CD36, and ERK1/2. CTNNAL1 and TXK reportedly activate the ERK1/2 signaling pathway.

IPA identified factors such as DDIT3, CTNNB1, RB1, BHLHE40, and STAT6, as key transcription factors in the hematopoietic process that function in the nucleus, along with ATF6 in the cytoplasm. This hematopoietic signaling pathway suggests that the identified candidate genes are not independent and are highly correlated.

## 4. Discussion

Hematopoiesis refers to the production of immature blood cells from hematopoietic stem cells (HSCs), which undergo differentiation mediated by a cascade and are dynamically processed in the BM into mature RBCs, WBCs, and PLTs circulating in the peripheral blood [25]. At a certain level, blood cells are eliminated via apoptosis or undergo clearance by the spleen [26]. Thus, the physiological homeostasis of the total amount of blood cells is maintained within a certain range. Under certain circumstances, exposure to toxic compounds, such as chemotherapeutic agents, alcohol, and benzene, typically induces suppression of BM development in a dose-dependent manner [27,28]. We investigated whether PHMG-p inhalation could induce systemic effects with an emphasis on hematopoiesis. In the present study, pulmonary fibrosis was induced 20 weeks after intratracheal PHMG-p exposure (Figure S1), accompanied by a significant alteration in the hematological laboratory parameters.

No significant differences in cellularity were detected following the BM biopsy. Furthermore, dysplastic or leukemic cells were absent in both groups, indicating that overall hematopoiesis was maintained and BM aplasia or hematologic malignancy was not induced. However, the number of megakaryocytes in BM decreased significantly. Based on these results, we speculated that PHMG-p could negatively impact the development/differentiation of PLT-producing megakaryocytes in the BM and eventually reduce PLTs in circulating peripheral blood, regardless of the liver and spleen volume. A statistically significant decrease in the proportion of monocytes to leukocytes in the PHMG-p-exposed group was detected (Table 1), suggesting that PHMG-p may also affect leukocyte differentiation via systemic circulation. Accordingly, although a two-week respiratory exposure to PHMG-p did not induce clinically evident hematologic disorders in rats, it could adversely impact hematopoietic differentiation in leukocytes and PLTs. Therefore, we performed RNA sequencing to examine transcriptional changes in the BM tissues of five rats selected per group.

According to the IPA prediction results, the expression of several hematopoietic signaling-related genes, such as activating transcription factor 6 (ATF6), DNA damage-inducible transcript 3 (DDIT3), catenin beta 1 (CTNNB1), and signal transducer and activator of transcription 6 (STAT6), would be inactivated in the BM tissue of Sprague–Dawley rats exposed to PHMG-p. ATF6 plays an important role in endoplasmic reticulum (ER) stress. Lopez et al. have shown that activated ER stress could induce megakaryocyte maturation and PLT release into the bloodstream [29]. Cleaved ATF6 translocates from the Golgi apparatus to the nucleus and regulates unfolded protein response (UPR)-related target genes, including *Xbp1* [30,31]. Thus, abnormal regulation of ATF6 may interfere with the differentiation of normal megakaryocytes, eventually leading to thrombocytopenia. In addition, interleukin (IL)-4, a Th2 cytokine, regulates the UPR pathway via STAT6 and STAT3, which is mediated through a synergistic response with IL-6 and IL-10 [32]. Wnt signaling, responsible for the formation and function of blood cells, plays an important role in proplatelet formation in megakaryocytes. β-catenin, encoded by CTNNB1, is a key transcription factor downstream of the Wnt signaling pathway [33]. DDIT3 is a lineage regulator that influences the direction of differentiation of multipotent cells and granulomonocytic progenitors during hematopoietic processes [34]. Aberrant methylation was found in the DDIT3 gene promoter in patients with chronic myeloid leukemia (CML) [35]. These findings suggest that PHMG-p affects PLT production as well as the overall hematopoietic process and could be a potential CML-causing factor.

Bipotent MEP cells, originating from the differentiation of myeloid stem cells, produce cells capable of forming megakaryocytes and RBCs [36]. Herein, we confirmed that, among the candidate genes, HPGDS, which exhibited the largest reduction (FC = −20.212, *p* = 0.036), was a representative gene highly expressed in the MEP lineage [24]. CREBBP is a histone acetyltransferase that plays an important role in hematopoiesis and is considered a tumor suppressor. Dysregulation of CREBBP activity (FC = −2.177, *p* = 0.020) is strongly associated with hematopoietic malignancies [37,38].

The reorganization of the cytoskeleton, such as actin and tubulin, of megakaryocytes is essential for the formation of proplatelets, an earlier stage of PLTs [39]. Rho GTPase is a key regulator of cytoskeletal rearrangement in cells such as megakaryocytes. Double deficiency of Rac1 and Cdc42 in megakaryocytes can reportedly induce morphological abnormalities in PLT and impair PLT function [40]. Surprisingly, ARHGAP4, which increased by 7.775-fold in the BM of the PHMG-p-exposed group, is a type of GTPase-activating protein, and Rho GTPase inactivation is promoted by GAP. In addition, we found that ARHGAP11A expression was upregulated by 2.892-fold. Conversely, GEF promotes an active conformation by altering the GDP-bound state to the GTP-bound state [41]. Our results revealed that expression of ARHGAP4 and ARHGAP11A increased by 7.775- and 2.892-fold, respectively, while ARHGEF3 expression decreased by 7.714-fold. These alterations in gene expression probably inhibit PLT production in megakaryocytes by downregulating Rho-like GTPase activity. CTNNAL1 is a coding gene for the alpha-catenin protein and plays a role in Rho signaling transduction. Furthermore, CTNNAL1 acts as a cytoskeletal scaffold protein and is widely known as a hematopoietic stem cell marker [42]. In addition, not only the decrease in the PLT production capacity of megakaryocytes, but also the decrease in the number of megakaryocytes in the BM itself is noteworthy. It is currently unknown whether the decrease in megakaryocytes numbers originates from maturation of megakaryoblasts of myeloid stem cells. However, since CTNNAL1 is a representative marker of HSC, its reduction has potential to have affected the differentiation pathway of HSC to myeloid stem cells [43].

Considering the homeostasis of the proportion between the two cell types constituting the leukocyte, upon exposure to PHMG-p, the total number of leukocytes did not change, but the proportion of granulocytes increased and agranulocytes decreased. Basophils and neutrophils belonging to granular leukocytes were increased, but not eosinophils, and the proportions of non-granular leukocytes (lymphocytes and monocytes) were decreased. Although the mechanism of these phenomenon is unknown, PHMG-p can be considered to interfere with the regulation of proportion homeostasis between the granulocytes and agranulocytes that make up leukocytes.

Taken together, we confirmed abnormalities in blood cell, and megakaryocyte counts in BM tissues of rats exposed to PHMG-p via intratracheal instillation. Abnormal gene transcriptional regulation in the BM was also confirmed. To the best of our knowledge, we first revealed that most altered candidate genes were strongly associated with hematopoietic processes and hematological malignancies.

## 5. Conclusions

Based on our findings, we suggest that exposure of the respiratory tract to PHMG-p, a major component of HDs, may exert a hematotoxic effect on hematopoiesis in the BM.

## Figures and Tables

**Table 1:** Peripheral blood tests focused on hematological markers in normal saline and PHMG-p-exposed rats (*n* = 15 rats/group).

Blood Test Items	Units	Normal Saline		PHMG-p		*z*-Scores	*p*-Value	Reference Interval	Ref.
		Mean	SD	Mean	SD				
WBC	10^3^/µL	6.50	1.46	6.70	1.91	−0.581	0.561	6.11 ± 3.11	[21]
Band Neutrophil	%	32.60	18.56	41.24	21.15	−1.141	0.254	32.48 ± 13.41	[22]
Lymphocyte	%	59.57	17.50	52.05	20.98	−0.892	0.373	63.24 ± 13.06	[22]
Monocyte	%	3.50	0.73	2.40	0.88	−2.970	0.003 *	3.49 ± 1.46	[23]
Eosinophil	%	2.57	1.75	2.45	1.81	−0.208	0.835	1.42 ± 1.94	[22]
Basophil	%	1.23	0.89	2.03	1.56	−1.746	0.081	1.16 ± 1.32	[23]
RBC	10^6^/µL	8.94	0.44	9.83	1.43	−1.556	0.120	8.79 ± 0.39	[23]
Hemoglobin (Hb)	g/dL	15.86	0.69	17.61	2.61	−1.973	0.049 *	15.69 ± 0.65	[23]
Hematocrit (Hct)	%	57.50	5.02	64.82	9.56	−2.261	0.024 *	46.19 ± 3.53	[23]
Platelet	10^3^/µL	928.67	142.71	703.07	179.99	−3.132	0.001 *	971.45 ± 126.77	[23]

Mean, standard deviation (SD), *z*-score, and *p*-value from the *t*-test or Mann–Whitney U test using raw data of the blood test items for the normal saline- and PHMG-p-instilled groups. Asterisk indicate a *p*-value of ˂0.05. PHMG-p, polyhexamethylene guanidine phosphate; RBC, red blood cell; WBC, white blood cell.

**Table 2:** Peripheral blood tests focused on biochemical markers in normal saline and PHMG-p-exposed rats (*n* = 15 rats/group).

Blood Test Items	Units	Normal Saline		PHMG-p		*z*-Scores	*p*-Value	Reference Interval	Ref.
		Mean	SD	Mean	SD				
Total protein	g/dL	6.25	0.51	5.79	0.31	−2.503	0.012 *	6.50 ± 0.52	[23]
Albumin	g/dL	3.87	0.38	3.74	0.16	−0.773	0.439	3.15 ± 0.21	[23]
BUN	mg/dL	22.07	3.73	26.40	7.60	−1.585	0.113	16.97 ± 2.78	[23]
Creatinine	mg/dL	0.59	0.14	0.67	0.13	−1.495	0.135	0.55 ± 0.07	[23]
CRP	mg/dL	<0.06	-	<0.06	-	-	-	-	-

Asterisk indicate a *p*-value of <0.05. BUN, blood urea nitrogen; CRP, C-reactive protein.

**Table 3:** Basic information for five rats per group selected based on platelet count.

Information	Units	Normal Saline		PHMG-p		*z*-Scores	*p*-Value
		Mean	SD	Mean	SD		
**Platelet count** *	**10^3^/µL**	**1068.6**	69.16	503.4	112.4	−2.611	**0.009** *
**Megakaryocyte count** *	10 HPF	204.4	33.49	133.8	40.33	−2.611	**0.009** *
Spleen weight	g	0.962	0.344	0.710	0.152	−1.149	0.251
Liver weight	g	17.98	3.199	15.36	4.717	−0.940	0.347
Body weight	g	507.0	10.42	496.4	63.40	−0.419	0.675
Spleen/Body weight	-	0.0019	0.0007	0.0014	0.0002	−1.149	0.251
Liver/Body weight	-	0.0354	0.0058	0.0305	0.0059	−1.149	0.251

Mean, standard deviation (SD), *z*-score, and *p*-value from the Mann–Whitney U test using raw data for the normal saline and PHMG-p-instilled groups. Asterisk and bold font indicate a *p*-value < 0.05. HPF, high-power field; PHMG-p, polyhexamethylene guanidine phosphate.

**Table 4:** List of 142 genes significantly altered in the bone marrow of rats exposed to polyhexamethylene guanidine phosphate (PHMG-p) via intratracheal instillation.

Symbol	Entrez Gene Name	Transcript ID	Fold Change	*p*-Value
Txk	TXK tyrosine kinase, transcript variant X1	NM_001024255	13.712	0.049
Ctnnal1	catenin alpha-like 1, transcript variant X1	NM_001106649	12.744	0.045
Ctsw	cathepsin W	NM_001024242	12.621	0.010
Hfe	hemochromatosis, transcript variant 1	NM_053301	9.440	0.044
Arhgap4	Rho GTPase-activating protein 4, transcript variant X1	NM_144740	7.775	0.038
Lactb	lactamase, beta, transcript variant X1	NM_001106833	7.416	0.021
Clcc1	chloride channel CLIC-like 1, transcript variant X1	NM_133414	5.885	0.036
Tyk2	tyrosine kinase 2, transcript variant X2	NM_001257347	5.764	0.018
Mrps23	mitochondrial ribosomal protein S23, transcript variant X1	NM_001108289	5.290	0.046
Slc11a1	solute carrier family 11 member 1, transcript variant X1	NM_001031658	4.966	0.027
Ap5z1	adaptor-related protein complex 5, zeta 1 subunit	NM_001037220	4.807	0.040
Gstz1	glutathione S-transferase zeta 1	NM_001109445	4.739	0.031
Rheb	Ras homolog enriched in brain	NM_013216	4.671	0.026
Lypd3	Ly6/Plaur domain-containing 3	NM_021759	4.667	0.046
Relt	RELT tumor necrosis factor receptor	NM_001108495	4.529	0.016
Lsm4	LSM4 homolog, U6 small nuclear RNA and mRNA degradation associated	NM_001106073	4.512	0.025
Ttpal	alpha tocopherol transfer protein like, transcript variant X1	NM_001106537	4.420	0.035
Ly6e	lymphocyte antigen 6 complex, locus E, transcript variant X4	NM_001017467	4.412	0.037
Taf1	TATA-box binding protein associated factor 1	NM_001191723	4.391	0.010
Nrros	negative regulator of reactive oxygen species, transcript variant X1	NM_001024995	4.388	0.008
Pank3	pantothenate kinase 3	NM_001108272	4.309	0.040
RGD1565784	RGD1565784	NM_001109028	4.267	0.013
Fchsd2	FCH and double SH3 domains 2, transcript variant X4	NM_001107539	4.158	0.017
Ogfr	opioid growth factor receptor	NM_053340	4.141	0.047
Eif3k	eukaryotic translation initiation factor 3, subunit K	NM_001106242	3.910	0.012
Mphosph6	M phase phosphoprotein 6	NM_001129881	3.820	0.046
Pnkp	polynucleotide kinase 3′-phosphatase, transcript variant X1	NM_001004259	3.714	0.016
Lgals5	lectin, galactose binding, soluble 5	NM_012976	3.688	0.035
Snx4	sorting nexin 4	NM_001127550	3.682	0.040
Fmo1	flavin-containing monooxygenase 1, transcript variant X2	NM_012792	3.622	0.035
Sik3	SIK family kinase 3	NM_001271216	3.603	0.010
Phkg2	phosphorylase kinase catalytic subunit gamma 2, transcript variant X2	NM_080584	3.537	0.047
Pip5k1b	phosphatidylinositol-4-phosphate 5-kinase type 1 beta	NM_001012743	3.505	0.041
Pld3	phospholipase D family, member 3, transcript variant X3	NM_001012167	3.429	0.005
Limk2	LIM domain kinase 2, transcript variant X1	NM_024135	3.397	0.017
Cirbp	cold-inducible RNA-binding protein, transcript variant X4	NM_031147	3.216	0.026
Pip5k1c	phosphatidylinositol-4-phosphate 5-kinase type 1 gamma, transcript variant X1	NM_001033970	3.186	0.004
LOC501110	similar to Glutathione S-transferase A1 (GTH1) (HA subunit 1) (GST-epsilon) (GSTA1-1) (GST class-alpha)	NM_001024361	3.137	0.023
Plcl2	phospholipase C-like 2	NM_001106880	3.121	0.041
Arhgap11a	Rho GTPase-activating protein 11A	NM_001168524	2.892	0.036
Polr2g	polymerase (RNA) II subunit G, transcript variant X1	NM_053948	2.867	0.042
Ptpn12	protein tyrosine phosphatase, non-receptor type 12	NM_057115	2.860	0.041
Emc2	ER membrane protein complex subunit 2	NM_001113785	2.845	0.014
Lrrc14	leucine-rich repeat containing 14, transcript variant X1	NM_001024354	2.834	0.001
LOC102549726	uncharacterized LOC102549726	NR_110713	2.803	0.017
Psmd9	proteasome 26S subunit, non-ATPase 9	NM_130430	2.797	0.010
Lsm2	LSM2 homolog, U6 small nuclear RNA and mRNA degradation associated, transcript variant 2	NM_001165922	2.742	0.016
Rcsd1	RCSD domain containing 1, transcript variant X1	NM_001108349	2.740	0.004
Cnot1	CCR4-NOT transcription complex, subunit 1	NM_001134840	2.720	0.014
Shisa5	shisa family member 5, transcript variant X1	NM_001006989	2.701	0.017
Eif6	eukaryotic translation initiation factor 6, transcript variant X2	NM_001037352	2.546	0.023
Junb	JunB proto-oncogene, AP-1 transcription factor subunit	NM_021836	2.518	0.045
Ube2i	ubiquitin-conjugating enzyme E2I, transcript variant X2	NM_013050	2.498	0.014
Kat6a	lysine acetyltransferase 6A	NM_001100570	2.430	0.028
Adck4	aarF domain-containing kinase 4, transcript variant X1	NM_001012065	2.429	0.006
C1qc	complement C1q C chain, transcript variant X1	NM_001008524	2.414	0.030
Zdhhc18	zinc finger, DHHC-type containing 18	NM_001039339	2.352	0.048
Snx5	sorting nexin 5	NM_001106518	2.316	0.004
Psmc3	proteasome 26S subunit, ATPase 3	NM_031595	2.252	0.002
Mmp9	matrix metallopeptidase 9	NM_031055	2.205	0.020
Abhd4	abhydrolase domain-containing 4, transcript variant X2	NM_001108866	2.162	0.008
Etfb	electron transfer flavoprotein beta subunit	NM_001004220	2.153	0.028
Cd74	CD74 molecule, transcript variant X1	NM_013069	2.132	0.005
Tor1aip2	torsin 1A interacting protein 2, transcript variant X3	NM_001165896	2.086	0.039
Ehd1	EH-domain containing 1	NM_001011939	2.082	0.048
Yipf3	Yip1 domain family, member 3, transcript variant X1	NM_001007801	2.046	0.046
Mxi1	MAX interactor 1, dimerization protein, transcript variant X1	NM_013160	2.028	0.037
Hpgds	hematopoietic prostaglandin D synthase, transcript variant X2	NM_031644	−20.212	0.036
Dr1	down-regulator of transcription 1, TBP-binding (negative cofactor 2)	NM_001011914	−19.743	0.014
Cabin1	calcineurin binding protein 1	NM_053575	−12.615	0.010
Ccnl1	cyclin L1, transcript variant X1	NM_053662	−11.615	0.009
Dtnb	dystrobrevin, beta, transcript variant X3	NM_001012191	−10.521	0.044
Tti1	TELO2 interacting protein 1, transcript variant X1	NM_001134619	−9.680	0.025
Zfp64	zinc finger protein 64	NM_001012093	−9.322	0.015
Atp5a1	ATP synthase, H+ transporting, mitochondrial F1 complex, alpha subunit 1, cardiac muscle	NM_023093	−9.259	0.047
Irf7	interferon regulatory factor 7, transcript variant X2	NM_001033691	−9.214	0.047
Ergic2	ERGIC and Golgi 2	NM_001024984	−8.686	0.014
Med13	mediator complex subunit 13	NM_001107035	−8.430	0.004
Lap3	leucine aminopeptidase 3	NM_001011910	−8.196	0.049
Arhgef3	Rho guanine nucleotide exchange factor 3, transcript variant X7	NM_001106061	−7.714	0.021
Pacsin2	protein kinase C and casein kinase substrate in neurons 2, transcript variant X12	NM_130740	−7.330	0.034
Rpp21	ribonuclease P/MRP 21 subunit	NM_001002831	−6.889	0.015
Tceb3	transcription elongation factor B subunit 3	NM_017103	−6.772	0.028
Ipo7	importin 7	NM_001107545	−6.583	0.041
Fam203a	family with sequence similarity 203, member A	NM_001007707	−6.267	0.032
Ascc3	activating signal cointegrator 1 complex subunit 3, transcript variant X3	NM_001277057	−6.076	0.001
Ipo13	importin 13	NM_053778	−5.873	0.003
Hsd17b1	hydroxysteroid (17-beta) dehydrogenase 1	NM_012851	−5.758	0.024
Ppib	peptidylprolyl isomerase B	NM_022536	−5.672	0.031
RGD735065	similar to GI:13385412-like protein splice form I	NM_199379	−5.650	0.023
Snx27	sorting nexin family member 27, transcript variant 1	NM_001110151	−5.484	0.020
Vat1	vesicle amine transport 1	NM_001033683	−5.279	0.031
Atp6v1e1	ATPase H+ transporting V1 subunit E1	NM_198745	−5.101	0.039
Rcor1	REST corepressor 1, transcript variant X1	NM_001305463	−5.030	0.009
Slc35c1	solute carrier family 35 member C1, transcript variant X1	NM_001107748	−4.994	0.031
Fbxo33	F-box protein 33, transcript variant X1	NM_001108023	−4.989	0.040
Tsc22d1	TSC22 domain family, member 1, transcript variant X1	NM_001109912	−4.892	0.023
Polb	polymerase (DNA) beta	NM_017141	−4.427	0.011
Tmem109	transmembrane protein 109, transcript variant X2	NM_001007736	−4.008	0.016
Klf16	Kruppel-like factor 16	NM_001127604	−3.909	0.042
Rpia	ribose 5-phosphate isomerase A	NM_001108632	−3.843	0.020
Tbcc	tubulin folding cofactor C	NM_001108200	−3.836	0.023
Morf4l2	mortality factor 4 like 2	NM_001007714	−3.746	0.033
Plagl2	PLAG1 like zinc finger 2, transcript variant X1	NM_001106528	−3.733	0.020
Pde4b	phosphodiesterase 4B	NM_017031	−3.718	0.019
Atg10	autophagy related 10	NM_001109505	−3.473	0.029
Nos1ap	nitric oxide synthase 1 adaptor protein	NM_138922	−3.455	0.005
Ncor1	nuclear receptor co-repressor 1	NM_001271103	−3.368	0.007
Slmo2	slowmo homolog 2	NM_001009543	−3.319	0.020
Ino80	INO80 complex subunit	NM_001261404	−3.199	0.002
Phyh	phytanoyl-CoA 2-hydroxylase	NM_053674	−3.183	0.046
Edc4	enhancer of mRNA decapping 4	NM_001033068	−3.141	0.034
Ccar1	cell division cycle and apoptosis regulator 1	NM_001108535	−3.052	0.008
Larp7	La ribonucleoprotein domain family, member 7	NM_001044290	−3.040	0.033
Cks1b	CDC28 protein kinase regulatory subunit 1B	NM_001135749	−3.020	0.026
Gps2	G protein pathway suppressor 2, transcript variant X1	NM_001017477	−3.014	0.008
Baz2a	bromodomain adjacent to zinc finger domain, 2A	NM_001107158	−3.006	0.027
Mrpl14	mitochondrial ribosomal protein L14, transcript variant 2	NM_001106890	−3.001	0.008
Kif2a	kinesin family member 2A	NM_053376	−2.953	0.011
Snx8	sorting nexin 8, transcript variant X1	NM_001105912	−2.936	0.030
Eif3f	eukaryotic translation initiation factor 3, subunit F	NM_001277302	−2.900	0.010
Ep400	E1A binding protein p400	NM_001107149	−2.898	0.008
Baz1a	bromodomain adjacent to zinc finger domain, 1A	NM_001170568	−2.887	0.002
Zrsr1	zinc finger (CCCH type), RNA-binding motif and serine/arginine-rich 1	NM_001017504	−2.883	0.046
Thg1l	tRNA-histidine guanylyltransferase 1-like, transcript variant X1	NM_001013966	−2.817	0.038
LOC294154	similar to chromosome 6 open reading frame 106 isoform a, transcript variant X1	NM_001039607	−2.644	0.010
Cdca8	cell division cycle associated 8, transcript variant X1	NM_001025050	−2.599	0.012
St3gal2	ST3 beta-galactoside alpha-2,3-sialyltransferase 2, transcript variant X3	NM_031695	−2.539	0.010
Psmd7	proteasome 26S subunit, non-ATPase 7	NM_001107426	−2.518	0.011
Ebf1	early B-cell factor 1, transcript variant X5	NM_053820	−2.513	0.007
Ublcp1	ubiquitin-like domain-containing CTD phosphatase 1, transcript variant X1	NM_001014117	−2.437	0.029
Zfp703	zinc finger protein 703	NM_001109425	−2.320	0.037
Luc7l3	LUC7-like 3 pre-mRNA splicing factor	NM_001108291	−2.217	0.015
Kmt2e	lysine methyltransferase 2E	NM_001100851	−2.201	0.009
Dhx15	DEAH-box helicase 15, transcript variant X1	NM_001191597	−2.189	0.029
Crebbp	CREB binding protein, transcript variant X2	NM_133381	−2.177	0.020
Metap2	methionyl aminopeptidase 2	NM_022539	−2.142	0.016
Srrm1	serine and arginine repetitive matrix 1, transcript variant X8	NM_001107986	−2.103	0.043
Dmtf1	cyclin D binding myb-like transcription factor 1, transcript variant X1	NM_053693	−2.090	0.029
RatNP-3b	defensin RatNP-3 precursor	NM_001079898	−2.074	0.032
Brd4	bromodomain containing 4	NM_001100903	−2.052	0.013
Tax1bp1	Tax1 binding protein 1, transcript variant X1	NM_001004199	−2.014	0.047

**Table 5:** Integrated gene analysis of top diseases and biological functions using Ingenuity Pathway Analysis.

Category	*p*-Value Range	No. of Molecules
Diseases and disorders		
Cancer	1.66 × 10^−2^–4.98 × 10^−8^	127
Organismal injury and abnormalities	1.67 × 10^−2^–4.98 × 10^−8^	129
Gastrointestinal disease	1.67 × 10^−2^–6.60 × 10^−6^	113
Hematological disease	1.65 × 10^−2^–1.30 × 10^−4^	50
Endocrine system disorders	1.38 × 10^−2^–2.44 × 10^−4^	101
Physiological system development and function		
Organismal survival	1.46 × 10^−2^–9.36 × 10^−7^	52
Connective tissue development and function	1.67 × 10^−2^–3.82 × 10^−5^	30
Skeletal and muscular system development and function	1.24 × 10^−2^–3.82 × 10^−5^	13
Tissue development	1.67 × 10^−2^–3.82 × 10^−5^	36
Hematological system development and function	1.67 × 10^−2^–4.49 × 10^−5^	39
Molecular and cellular functions		
Cell cycle	1.61 × 10^−2^–4.31 × 10^−7^	43
Gene expression	1.55 × 10^−2^–3.56 × 10^−6^	45
Post-translational modification	5.53 × 10^−3^–7.34 × 10^−5^	4
DNA replication, recombination, and repair	1.52 × 10^−2^–9.30 × 10^−5^	15
Cellular development	1.64 × 10^−2^–1.28 × 10^−5^	51

**Table 6:** Top five specific functions for ‘hematological disease’ and ‘hematological system development and function’ in Table 5.

Disease or Bio Function Annotation	*p*-Value	Molecules	# Mol.
Hematological disease			
Abnormal morphology of bone marrow	1.30 × 10^−4^	CREBBP, EBF1, EP400, KAT6A, TYK2	5
Bone marrow neoplasm	7.41 × 10^−4^	ATP5F1A, ATP6V1E1, CCNL1, CD74, CKS1B, CREBBP, CTSW, DHX15, DTNB, EDC4, EP400, IPO7, JUNB, KAT6A, KMT2E, LY6E, LYPD3, MMP9, NCOR1, PDE4B, PLAGL2, PLCL2, POLB, PSMD9, TAF1, TYK2, UBE2I, ZFP64	28
Central nervous system leukemia	1.09 × 10^−3^	MMP9, POLB	2
T acute lymphoblastic leukemia	1.35 × 10^−3^	CREBBP, EBF1, NCOR1, POLB, TAX1BP1, TYK2	6
Incidence of lymphoma	1.74 × 10^−3^	CD74, DMTF1, INO80, JUNB, MXI1, TYK2	6
Hematological system development and function			
Morphology of lymphoid tissue	4.49 × 10^−5^	BRD4, CD74, CKS1B, CREBBP, GSTZ1, HPGDS, IRF7, KAT6A, KMT2E, MXI1, NCOR1, SLC35C1, SNX27, ST3GAL2, TYK2	15
Leukopoiesis	1.28 × 10^−4^	C1QC, CD74, CREBBP, DMTF1, EBF1, EIF6, GPS2, IRF7, JUNB, KMT2E, NCOR1, NRROS, PIP5K1C, PLCL2, POLB, PTPN12, RHEB, TXK, TYK2	19
Development of hematopoietic cells	4.23 × 10^−4^	CD74, CREBBP, EBF1, EIF6, KMT2E, MMP9, NCOR1, RCOR1, TYK2	9
Conjugation of T lymphocytes	6.41 × 10^−4^	PIP5K1C, TXK	2
Quantity of erythroid cells	1.36 × 10^−3^	CREBBP, GPS2	2

## Data Availability

All data generated or analyzed during this study are included in this published article.

## Supplementary Materials

The following are available online at https://www.mdpi.com/article/10.3390/toxics10110694/s1, Figure S1: Rat lung tissue sections stained with hematoxylin and eosin (100×); Table S1: Basic information of 30 rats (15 normal saline and 15 PHMG-p instilled groups).

## Abbreviations

PHMG-p: polyhexamethylene guanidine phosphate; HDs, humidifier disinfectants; BM, bone marrow; PLT, platelet; IPA, Ingenuity Pathway Analysis; Hb, hemoglobin; Hct, hematocrit; WBC, white blood cell; RBC, red blood cell; BUN, blood urea nitrogen; HPF, high-power field; FC, fold change; GEF, guanine nucleotide exchange factor; MEP, megakaryocytic-erythroid progenitor; ER, endoplasmic reticulum; UPR, unfolded protein response; CML, chronic myeloid leukemia; HSC, hematopoietic stem cell.
