# Supplementary material for: Hematotoxic Effect of Respiratory Exposure to PHMG-p and Its Integrated Genetic Analysis
Source: Toxics. 2022 Nov 16;10(11):694. doi: 10.3390/toxics10110694 (PMC9693004; doi:10.3390/toxics10110694)
Supplement: Supplementary file 1 [file toxics-10-00694-s001.zip › toxics-2007790-supplementary.pdf]

toxics

Supplementary Materials: Hematotoxic Effect of Respiratory Exposure to PHMG-p and Its Integrated Genetic Analysis

Hwa Jung Sung, Sang Hoon Jeong, Ja Young Kang, Cherry Kim, Yoon Jeong Nam, Jae Young Kim, Jin Young Choi, Hye Jin Lee, Yu Seon Lee, Eun Yeob Kim, Yong Wook Baek, Hong Lee and Ju Han Lee

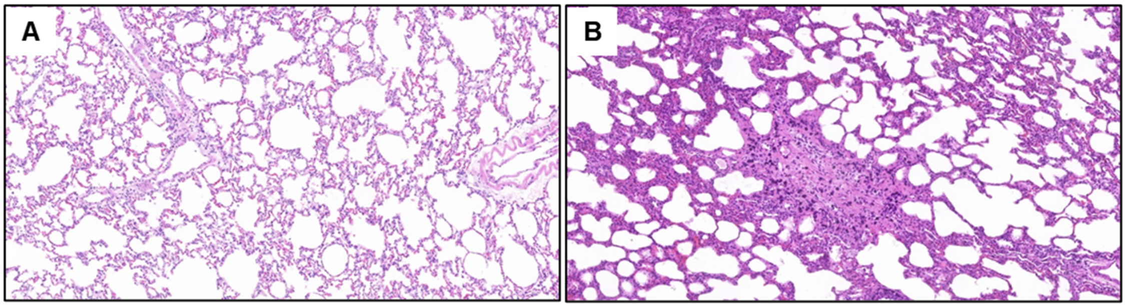

Figure S1. Rat lung tissue sections stained with hematoxylin and eosin (100×). (**A**) Lung tissue obtained from vehicle control (normal saline) group showed normal architecture with no fibrosis. (**B**) Lung tissue obtained from PHMG-p intratracheal instilled group represented pulmonary fibrosis with interalveolar septum thickening and inflammatory cells infiltration.

Table S1. Basic information of total 30 rats (15 normal saline and 15 PHMG-p instilled group).

Information	Units	Normal Saline		PHMG-p		*z* Scores	*P* Value
		Mean	SD	Mean	SD		
**Platelet count***	10^3/uL	928.7	142.7	703.1	180.0	3.804	**0.001***
Megakaryocyte count	10 HPF	173.0	31.99	151.1	30.40	1.925	0.064
**Spleen weight***	g	1.061	0.287	0.779	0.114	-2.924	**0.003***
Liver weight	g	18.31	3.637	16.00	3.372	1.804	0.082
Body weight	g	506.3	29.20	507.3	41.50	-0.071	0.944
**Spleen/Body weight***	-	0.0021	0.0006	0.0015	0.0002	-2.675	**0.007***
**Liver/Body weight***	-	0.0360	0.0061	0.0313	0.0048	2.345	**0.026***

1) Platelet count, megakaryocyte count, liver weight, body weight and liver/body weight from the T test, spleen weight and spleen/body weight from the Mann-Whitney U test using raw data for the normal saline and PHMG-p instilled group.
2) Asterisk and bold font indicate a p value less than 0.05.
